# Extending neurological care to a developing world

**DOI:** 10.4103/2152-7806.69381

**Published:** 2010-09-16

**Authors:** Melvin L. Cheatham

**Affiliations:** Clinical Professor of Neurosurgery, UCLA David Geffen School of Medicine, Los Angeles, CA, USA

Most of the specialty areas of Neurological Science have developed over the course of the past 70 years. This has come about with the advent of angiography followed by nuclear scanning, computed tomography (CT), magnetic resonance imaging (MRI), and positron emission tomography (PET) scanning. The operating microscope and surgical loops have brought us microneurosurgery. Various instrumentation measures provide methods of spine stabilization. The joint efforts of various specialties have refined surgery at the skull base. The electronic age and computers have provided us the ability to communicate and educate on a worldwide basis. Yet, neurological care for people in developing countries is largely minimal or non-existent. The question is: “What can we do to extend neurological care to the developing world?

Imagine having to render neurological patient care without the benefit of CT and MRI scanning, catheter angiography, intracranial pressure monitoring, Decadron, Mannitol, the operating microscope, computers, intensive care units, and the wide array of medications available to us today. Many physicians in developing countries do not have to “imagine” what it would be like to care for patients without these essentials of modern-day neurological practice.

Perhaps surprising to many readers of *Surgical Neurology International* to know that those of us senior enough in years as Neurologists and Neurosurgeons remember a time when none of the “essentials” for practicing our professions, as listed above, were available. For me, this was a journey which began more than 50 years ago when, as an intern on the Neurosurgical Service at the University of California in San Francisco, none of these present-day essentials in our medical specialty were available.

Looking back at what was available in the medicine in those 50 or more years ago, the first Intensive Care Unit (ICU) had just been envisioned. At that time, patients requiring close monitoring (what we now identify as “intensive care”) were either kept in the hospital’s recovery room or placed in a private room equipped with a suction machine, oxygen, perhaps an EKG monitor, and “round-the-clock” nurses.

Then, in 1953, it was Dr. Bjorn Aage Ibsen, who, in a converted student nurse classroom in Kommunehospitalet Hospital in Copenhagen, set up what became the world’s first Medical/Surgical ICU. Hospitals in medical centers around the world slowly followed the lead of Dr. Ibsen, eventually realizing the truly life-saving benefits offered by the ICUs.

In the late 1950s, cerebral angiography was being done primarily by needle puncture of the carotid and vertebral arteries using Cournand needles, with stacks of X-ray cassettes being “pulled” by hand to obtain sequence angiograms. Ventriculograms, pneumoencephalograms, nuclear brain scans, evidence of midline shifts suggested by ultrasound, exploratory burr holes and sometimes craniotomies, all these methods were used in trying to diagnose intracranial pathology.

In 1973, Professor Geoffrey N. Hounsfield at EMI Ltd. (Hays, England) developed the technology for computer assisted tomography, and with the birth of the EMI scanner in 1974, neurological diagnosis experienced a giant leap forward. The CT images were very much lacking in definition, but for the first time it was possible to “see inside the hard-boney box” that is the skull; this made possible because of computer technology.

In 1979, Professor Hounsfield was made a Nobel Laureate in Medicine. Through his highly innovative scientific work, and that of others who further developed his pioneering efforts, the highly detailed and informative brain, spine and other CT scanning capabilities of today were made possible. Similarly, MRI has been progressively refined to a point where visualizing both normal anatomy and areas of pathology has reached amazing levels of detail. Even functional activity in the brain can now be imaged, and in three dimensions.

Perhaps no other addition to the way neurosurgical operations are performed has contributed so much to the level of neurosurgical excellence that we know today like that contributed by the operating microscope. Long used by Ophthalmologists, ENT and Plastic Surgeons, it was the work during the late 1950s and early 1960s of Professors Robert Rand and Peter Jannetta at UCLA, Professor M. Gazi Yaşargil in Zurich, and the pioneering efforts of others, which launched microneurosurgery as we know it today.

Another critically important development in microneurosurgical capabilities came through the cooperative efforts of neuroanatomists, neuroradiologists, neurosurgeons, ENT surgeons, Ophthalmic surgeons and Plastic surgeons. This came as a result of these various surgical specialties sharing areas of surgical expertise, and even operating as teams, utilizing the best of their levels of expertise. It also came through a shared awareness of the importance of a detailed knowledge of neuroanatomy and the need to utilize this knowledge in neuroradiologic diagnosis and treatment.

The final and very significant addition to the enormous advancements that have come in the fields of neurological diagnosis and treatment over the past 40 years has resulted from the computer and electronic age, and it has been the technology necessary for communicating medical information. The ability to develop, teach, share and disseminate visual and written medical and surgical information, and to do so instantaneously and globally, has truly propelled medical science to previously unimaginable heights.

It is beyond the scope of this editorial article to even begin to identify the multitude of exciting and highly meaningful developments that have come through the computer and electronic generation over the past 40 years. As highly trained and highly skilled neurological specialists, we can probably feel comfortable in saying “we probably haven’t seen anything yet!” The ever newer and more exciting advances in medical science that will likely become available to us in the months and years ahead will almost certainly be in the realm of “the unimaginable,” as have been the developments of the scientific generation in the recent past.

It is precisely with our awareness of this fact that the new journal “*Surgical Neurology International*” brings fresh excitement. The ability now exists in disseminating the information worldwide, at any moment, and in any location in the entire world where there is Internet access, neurosurgical information can be shared.

In a sense, we, as neurological specialists, now find ourselves standing “near the top of the mountain” in terms of the patient care we can give in our field of endeavor. We also find ourselves prepared and equipped through the mechanism of sharing written and visual information now available to us through *Surgical Neurology International*, to always work toward further achieving higher levels of excellence in what we do.

May it be our goal always to do this, never being satisfied with the miracles that are ours today, but always to pursue the research and investigative efforts that can bring to us and our patients the miracles of tomorrow.

Before writing the words that will bring this editorial article to a close, and certainly before patting ourselves on the back for having achieved the goals we have reached as neurological specialists in the past, there is something very important that cannot be ignored or forgotten. In medical centers in developed countries around the world, levels of neurological diagnosis and treatment do seem to be near the mountain top. Unfortunately, care of this kind for the majority of people in the world today is likely non-existent.

Here again, *Surgical Neurology International* and the top-tier of Neurological and Neurosurgical specialists who are so strategically positioned worldwide, can become the network through which neurological treatment is brought to people in underdeveloped countries where it is not currently available. A number of editorial articles on *Physician Volunteerism* have already appeared in the medical literature. Many physicians are currently spending some of their time doing this kind of work and it is likely that many others will want, with a little encouragement, to do so.

While some specialists in the neurological sciences may feel overqualified for doing meaningful volunteer work in remote hospitals in developing countries, many might well be willing to go to developing countries for the purpose of teaching national doctors in those countries. It has been shown repeatedly that through short-term volunteer medical teaching visits of this kind, even volunteers with professorial rank tend to return home with a vision of obtaining donations of medical equipment, and for organizing medical teams, then returning for more short-term work. Also, for older neurosurgeons, working in developing countries is similar to what they did in the early years of their training before many advances outlined above occurred.

We have come a long way since the time of Drs. Harvey Cushing, Walter Dandy and others who were the early pioneers in our specialty. Those of us who have lived and practiced our specialty through this past half-century have certainly been blessed in having done so during what many be referred to as “The Golden Years of Medicine.” Even so, the “Golden Years” of another generation are now being lived and *Surgical Neurology International* has been launched. It is an instantly accessible, state-of-the-cutting-edge-of-the-art neurological specialty journal and its mission is to “Make a Difference” in health care delivery in the specialties of neurological science. There can be no greater calling upon us as doctors of medicine than to “Make A Difference” in the lives of our patients.

